# Fabrication and Characterization of Novel Electrothermal Self-Healing Microcapsules with Graphene/Polymer Hybrid Shells for Bitumenious Material

**DOI:** 10.3390/nano8060419

**Published:** 2018-06-09

**Authors:** Xinyu Wang, Yandong Guo, Junfeng Su, Xiaolong Zhang, Yingyuan Wang, Yiqiu Tan

**Affiliations:** 1 School of Mechanical Engineering, Tianjin University of Commerce, Tianjin 300134, China; 2 Department of Polymer Material, School of Material Science and Engineering, Tianjin Polytechnic University, Tianjin 300387, China; 1730021103@stu.tjpu.edu.cn (Y.G.); 15900294905@163.com (X.Z.); 3 School of Transportation Science and Engineering, Harbin Institute of Technology, Harbin 150090, China; alienwyy@sina.com (Y.W.); yiqiutan@163.com (Y.T.)

**Keywords:** nanohybrid, self-healing, microcapsule, graphene, bitumenious material

## Abstract

Self-healing bituminous material has been a hot research topic in self-healing materials, and this smart self-healing approach is a promising a revolution in pavement material technology. Bitumen has a self-healing naturality relating to temperature, healing time, and aging degree. To date, heat induction and microencapsulation rejuvenator are two feasible approaches, which have been put into real applications. However, both methods have disadvantages limiting their practical results and efficiency. It will be an ideal method combining the advantages and avoiding the disadvantages of the above two methods at the same time. The aim of this work was to synthesize and characterize electrothermal self-healing microcapsules containing bituminous rejuvenator with graphene/organic nanohybrid structure shells. The microcapsules owned electric conductivity capability because of the advent of graphene, and realized the self-healing through the two approaches of heat induction and rejuvenation. The microcapsule shells were fabricated using a strength hexamethoxymethylmelamine (HMMM) resin and graphene by two-step hybrid polymerization. Experimental tests were carried out to character the morphology, integrity, and shell structure. It was found that the electric charge balance determined the graphene/HMMM microstructure. The graphene content in shells could not be greatly increased under an electrostatic balance in emulsion. X-ray photoelectron spectroscopy (XPS), Energy dispersive spectrometer (EDS), Transmission electron microscope (TEM) and Atomic force microscopy (AFM) results indicated that the graphene had deposited on shells. TGA/DTG tests implied that the thermal decomposition temperature of microcapsules with graphene had increased to about 350 °C. The thermal conductivity of microcapsules had been sharply increased to about 8.0 W/m^2^·K with 2.0 wt % graphene in shells. At the same time, electrical resistivity of microcapsules/bitumen samples had a decrease with more graphene in bitumen.

## 1. Introduction

The function of self-healing is one of the most basic characteristics of nature [1,2,3]. Self-healing materials are believed to be the functional systems with inspiration coming from the evolutionary optimization. These materials have the capability of healing themselves and regenerating their functions when damaged by external destruction. Self-healing materials have been divided into two categories according to their repair mechanism and whether they were implanted with external repair agents [4]. One is called the intrinsic self-healing material, which can be obtained through different chemical reaction approaches, such as photo inducement, recombination of chain-end, molecular interdiffusion and reversible bond formation [5]. Another is the extrinsic self-healing material, which may not possess an intrinsic self-healing capability or has a relatively weak self-healing capability. In order to achieve the self-healing function, these materials need external healing components by deliberately embedding microcapsules and hollow fibers containing self-healing agents [6].

In recent years, self-healing concept was introduced into the field of engineering materials and construction materials, such as composites, biomaterials, concrete, and asphalt [7]. It is worth mentioning that self-healing bitumen has attracted increasing attention in self-healing materials because this smart self-healing technology is potentially revolutionary for pavement materials. Predictably, self-healing materials will supply a transmission of ideas to road maintenance simultaneously by decreasing the cost and increasing the life of pavement [8]. Self-healing bitumen can reduce the dosage of new materials in pavement maintenance, decrease traffic blockage during maintenance, decrease pollutant and greenhouse gases emissions, and elevate road safety and lifespan [9]. In other words, self-healing technology will enhance the level of intelligence of roads for the future. At present, bitumen pavement design follows the principles of enhancing performance, increasing durability, and improving load-carrying capability. With the progress of intelligent science and technology, bitumen pavement design may motivate efforts to accomplish the goal of allowing roads repair themselves to a certain extent from their original state [10]. 

In general, the self-healing capability of asphalt pavement can be promoted depending on the supplementary role of external materials. In order to obtain the above goal, these additives must survive and prevail in the harsh conditions during treatment process and service life in asphalt [11]. To date, a literature review shows that two methods are considered as the effective approaches for self-healing bitumen materials. The first one is called the heat induction method, which has gained popularity in self-healing bitumen research [12,13]. Electrical conductive fibers and fillers added into bitumen pavement as well as the heat produced by electric current could enhance the self-healing capability of bitumen. At the moment when the conductive bituminous material was put under a coil, the electromagnetic field immediately induced an electric current flowing along the conducting coils of steel fibers. [14]. It was found that the electrical capability of bitumen samples significantly depend on the size, shape, and type of fibers. An alternating current in the coils generated an alternating electromagnetic field. The electrical-thermal process softened the bitumen. The flowing bitumen filled the gap of cracks and healed the cracks through self-viscoelasticity. This process can be operated repeatedly when the cracks grew appearance. Interestingly, another method is the usage of microencapsulated oily rejuvenator [15]. The mechanism of this approach has been repotted systemically in previous works [15,16,17]. Several microcapsules will be punctured by the tip-stress of a microcrack in it’s the propagation path. The broken microcapsules then discharged oily rejuvenator. With the action of a capillary, the rejuvenator rapidly filled the interspaces of the microcracks. The small molecules of rejuvenator can diffuse into and soften the bituminous molecules in binders. The viscous flow will facilitate a healing process and prevent a further propagation of this crack [16]. The self-healing processes mentioned above are also repeatable as a multi-self-healing has been observed in bitumen mixing with microcapsules containing rejuvenator. In particular, the microencapsulated waste cooking oil (WCO) was recycle-used as self-healing additive in asphalt [17]. This idea has potential environmental value and technological value. Both technologies have been reported to be the most feasible self-healing methods for bituminous materials. At the same time, both have exposed their disadvantages during a transition from laboratory researches to real applications [12,16]. For the heat induction method, it encounters a fatal flaw that the heat accelerates the aging process of bituminous materials. More cracks occur under such a repeated heat induction. The steel fibers will loss their conductivity because of the unavoidable corrosion. It is inconvenient to carry out the healing process using dedicated equipment. The healing process consumes a large amount of energy using metal fibers. It must be pointed out that the steel fibers extending out of road surface is a great danger to the safety of traffic because steel fibers are a possibility of a flat tire. The microencapsulation rejuvenator method is an ideal way to healing the microcracks in bitumen. However, the self-healing speed is not satisfied with the real application [18]. At the same time, cracks with a larger size cannot be healed using microcapsules and heat is necessary to melt the bitumen and eliminate the cracks [15]. 

Inspired by the above analysis, it will be an ideal method to combine the advantages of the above two methods. We can image that the microcapsules containing rejuvenator with electric conductivity capability will realize the self-healing through two approaches: heat induction and rejuvenation. The advent of graphene provides the possibility for the realization of this assumption. Graphene is the first two-dimensional (2D) atomic crystal exhibiting special properties, such as high stiffness, high electrical conductivity, high thermal conductivity, and high barrier property [19,20]. These properties suggest that graphene could combine a wide variety of materials for very wide applications. For examples, graphene has been used to form composites with polymers [21,22,23], metals [24,25,26], and ceramics [27,28], which have displayed special combination performances. Enlightened by the above applications, new microcapsules can be prepared as potential opportunities for various applications, such as microsensors, microreactors, and energy container [29].

From the first reported mention of self-healing composites in the literature, a conventional method was obtained and extensively investigated by mixing microcapsules containing healing agents or chemical materials in polymer materials [30]. To date, the microcapsule-based system is arguably the most popular that has been used in many self-healing materials, such as synthetic polymers [31,32], biopolymers [33], asphalt [34], and concrete [35]. Accordingly, while damage triggers cracks in a matrix material, microcapsules consequently release their microencapsulated liquid healing agent into the microcrack cavity. In order to deposit the graphene on microcapsules, a polymer material is needed to form inorganic/organic composite shells. Microcapsule shells must withstand the competent processing conditions of the matrix composite, and maintain good combination with the polymer matrix ensuring the shells fracture by cracks in composites. Initially, dicyclopentadiene (DCPD) was encapsulated as healing agent by urea-formaldehyde (UF) [36,37] and/or epoxy [38,39]. In addition, melamine-formaldehyde [40], methyl melamine-formaldehyde (MMF) [41], and polyurethane [42] materials were as well successfully applied to fabricate different self-healing microcapsules. All these reported polymers have a good encapsulation effect. Graphene oxide is easy to have a chemical reaction with other polymers. However, graphene has an inorganic material with a sheet structure without any organic groups. Therefore, it is difficult to deposit graphene on shells of microcapsules. Physical absorption or physical entanglements are methods to lead the graphene depositing on shells. A higher cross-linking polymer structure may help to realize the above purpose. In this study, hexamethoxymethylmelamine (HMMM) resin was selected as the polymeric shell material, which was compounded together with graphene to form the shells of microcapsules. HMMM resin is a highly methylated melamine formaldehyde resin, which has more bonds improving the capability of dissolving in water. At the same time, a highly cross-linked structure helps the graphene to deposit on shells.

The aim of this work was to conduct a preliminary investigation of electrothermal self-healing bituminous composite material using microcapsules containing rejuvenator. These microcapsules were fabricated with a graphene/HMMM hybrid structure by a two-step self-assembly method with the help of macromolecules entanglement and electrostatic adsorption. HMMM carried more positive charges and graphene was negatively charged in an alkaline environment. Graphene was successfully introduced into the microcapsule shells, which greatly improved the electrical conductivity and thermal conductivity of the shells. Morphology, chemical structure, heat properties, and electric conductivity had been systemically investigated based on microcapsule samples with different components of graphene. In a microcapsules/bitumen composite system, the self-healing occurred when a low voltage was added to the material forming a closed loop to generate heat. The heat greatly enhanced the self-healing capability of materials.

## 2. Experimental

### 2.1. Materials

Graphene was commercial product supplied by Tuling Co., Ltd. (Shenzhen, China). Oily rejuvenator was used as the self-healing agent (0.905 g/cm^3^, 4.24 Pa·s, Tianjin Sinogo. Co., Ltd., Tianjing, China). Prepolymer hexamethoxymethylmelamine (HMMM) was used as the polymeric shell material supplied by Tianjin Sinogo. Co., Ltd. (solid content of 98.0%, Tianjing, China). A copolymer of styrene maleic anhydride (SMA) was purchased as a dispersant (Hercules, CA, USA). Bitumen was supplied by Qilu Petrochemical of China. A 40/50 (penetration grade) bitumen sample was artificially manufactured with a 80/100 (penetration grade) bitumen sample using a thin film oven process.

### 2.2. Prepare of Microcapsules with Graphene

**Figure 1:**
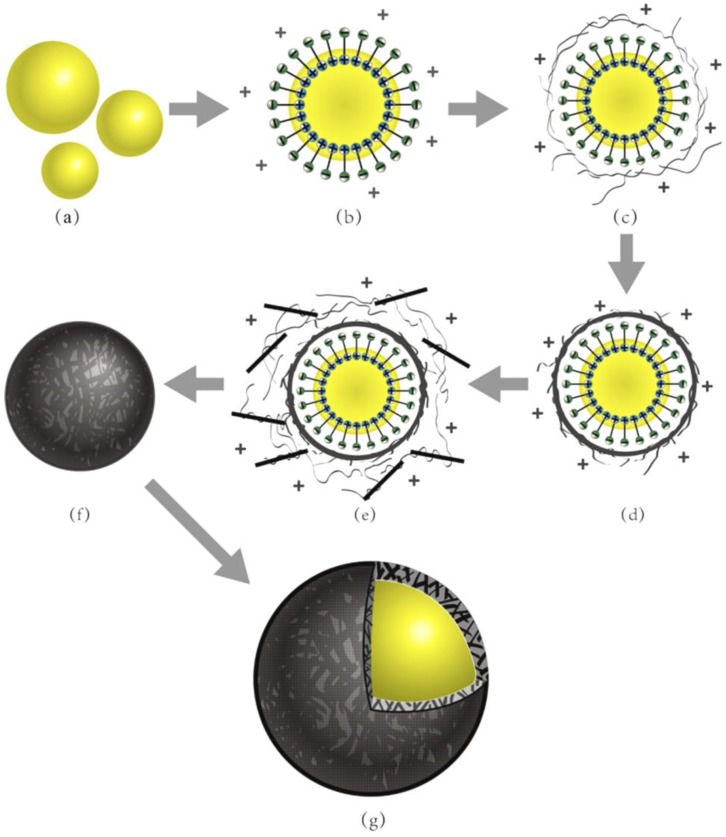
Illustration of self-healing microcapsules containing rejuvenator fabrication process by a two-step self-assembly method, (**a**,**b**) rejuvenator emulsified by SMA molecules, droplets with negative charge; (**c**) HMMM prepolymer added dropwise and attached to the surface of the droplets, droplets with positive charge; (**d**) the HMMM prepolymer cross-linked in the first step of the polymerization; (**e**) the mixture of HMMM prepolymer and graphene added dropwise, HMMM prepolymer and graphene absorbed on core droplets by charge; (**f**) microcapsules formed through a second step polymerization process; and (**g**) a three-dimensional anatomical structure of one self-healing microcapsule with a graphene/HMMM shell.

Figure 1 illustrates a preparation process of microcapsules containing rejuvenator with graphene/HMMM hybrid shells by a special two-step self-assembly method. The whole process was divided into four steps: (1) SMA powder was mixed with 50 °C water. The pH value of this mixture was adjusted by a NaOH solution to 10. Oily rejuvenator emulsified was under a vigorous stirring in the above surfactant solution for 10 min (Figure 1a,b). (2) HMMM prepolymer was then added dropwise into the above mixture emulsion accompanying a 400 r·min^−1^ stir (Figure 1c,d). The temperature of emulsion was slowly changed to 50 °C. (3) A mixture of HMMM prepolymer and graphene was again supplied with a stirring of 300 r·min^−1^. Then the temperature was increased to 80 °C with a speed of 3 °C·min^−1^. After 2 h of chemical reaction, the temperature was regulated to 20 °C (Figure 1e,f). (4) Finally, the microcapsules were filtered from the solution and washed with pure water (Figure 1g). It was critical that the prepolymer or prepolymer mixing with graphene was added dropwise into the emulsion at a slow speed of 0.5 mL·min^−1^. The reason is that this dropwise method could realize the polymerization with a slow speed forming a perfect shell structure [17]. Meanwhile, the graphene and prepolymer also had enough time to deposit on shells with the help of electrical charges.

### 2.3. Characterization of Microcapsules

The state of the materials in emulsion was observed by a biological microscope (Boshi Co. Shenzhen, China). A SEM (FEI Nanosem 430, Hillsboro, OR, USA) was used to analyze the surface morphologies of dried microcapsules. The mean size of microcapsule samples were tested by a particle size distribution instrument (JHY-1076, Jinheyuan, Xiamen, China). In order to measure the shell thickness, an ultramicrotomy was applied. About 10 g of microcapsules was blended with gelatin solution (30 wt %) forming a composite sample. The dried sample was carefully cut to obtain the cross-section slides by an ultramicrotome (FC7-UC7, Leica, Wetzlar, Germany). The thickness values of various shells were measured under a microscope with an average value of fifty microcapsules. Fourier transform infrared spectra were used to analyze the chemical structures of microcapsule samples (FT-IR, NICOLET Magna 750, Waltham, MA, USA).

### 2.4. Microstructure of Graphene in Shells

The carbon structures of microcapsules were characterized by an X-ray photoelectron spectroscopy (XPS, ESCALAB, Thermo Fisher, Waltham, MA, USA). The elements ratios (C, N, and O) were measure using an energy dispersive spectrometer (EDS, APOLLO XL, EDAX, Mahwah, NJ, USA). Transmission electron microscope (TEM) was utilized to analyze the graphene state in shells (Hitachi HT7700, Tokyo, Japan). Atomic force microscopy (AFM, S5500, Agilent, Santa Clara, CA, USA) examination was carried out at room temperature using silicon cantilevers.

### 2.5. Thermal Resistance of Microcapsules

The thermal resistance characteristics of microcapsules were tested using thermogravimetric analysis (TGA, SDT-2960, Dupont, Wilmington, NC, USA) with a scanning rate of 5 °C·min^−1^ under a N_2_ protection [38].

### 2.6. Electric Conductivity Measurement

The electric conductivity of microcapsule powder was measured by a powder electrical resistivity instrument (FT-310A, Ningbo KW Instrument Co., Ltd., Ningbo, China). The test method met the criterion of ISO 11713-2000. The instrument had an accuracy of 0.01 μΩ∙mm. The bitumen (40/50) was blended with various contents of microcapsules at 160 °C stirring with a speed of 300 r·min^−1^. The electric conductivity of the composite samples was tested using a four-point probe electrical resistivity instrument (FT-330, Ningbo KW Instrument Co., Ltd., Ningbo, China). The test method met the criterion of ASTM F84. The instrument had an accuracy of 0.01 μΩ∙mm.

### 2.7. Thermal Conductivity Measurement

The thermal conductivity measurement was carried out by using an instrument (JB, Shanghai, China) was used to test the thermal conductivity of samples according to measurement standard of ASTM E-1530. A sample was put between two polished aluminum (Al) plates holding with a constant compress stress. The Al plates were controlled at two different temperatures and the lower one was part of a transducer for calibrated heat flow. Because of the temperature differential, the heat flowed from one to another plate forming an axial temperature gradient. Temperature sensors monitored the temperature drop through the sample. The various temperatures of sample were recorded along with the output from the heat flow transducer after reaching a temperature equilibrium state. The thermal conductivity then was calculated by utilizing these temperature data and the sample thickness values. Thermal conductivity values are calculated by Equation (1),

(1) $$H=\lambda A\frac{T_{1}-T_{2}}{s}$$

where *T*_1_ and *T*_2_ are temperature values of two plates, *A* is the heat transition area, *s* is the thickness of sample, λ is the thermal conductivity, *H* is the heat flux.

## 3. Results and Discussion

### 3.1. Morphologies and Geometrical Characteristics

In this study, microcapsules were fabricated using HMMM prepolymer as the shell material through a two-step polymerization. In previous works, it had been found that both of melamine-formaldehyde (MF) resin and MMF resin were successfully applied to fabricate microcapsules with a compact shell structure [17,41]. Core droplets were ultimately separated through the regulation of hydrolyzed SMA molecules. With the help of disperse agent of SMA, core droplets were formed in emulsion. Then single monomer or prepolymer was absorbed on the droplet surfaces by electrostatic interactions [37]. Interestingly, inorganic nano-particles were also adhered on the droplets surface by promoting effects of chain entanglement electrostatic attraction [16,43]. At an equilibrium point, MMF-prepolymer molecules were cross-linked to form the nano-inorganic/organic shells under a high temperature and a proper pH value [38]. The surface morphology, mean size and shell thickness were normally defined as the three basic parameters of microcapsule product, which was controlled by regulating the polymerization process including the emulsion-stirring rate, prepolymer adding speed and core/shell weight ratio [41].

**Figure 2:**
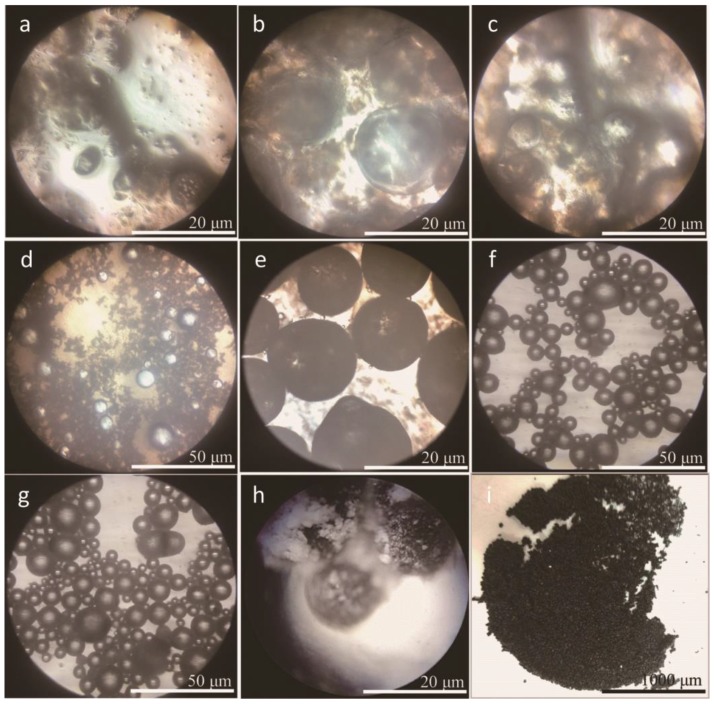
Optical microscope morphologies of self-healing microcapsules with 2% content of graphene in shells fabricated by a two-step polymerization, (**a**) oily rejuvenator emulsified by hydrolyzed SMA; (**b**) HMMM prepolymer added in emulsion; (**c**,**d**) the first-step polymerization; (**e**) HMMM prepolymer and graphene mixture added again; (**f**,**g**) the second-step polymerization; (**h**) the filtered microcapsules with impurity on shells; and (**i**) the washed and dried microcapsules.

Based on previous works, a novel two-step polymerization method was applied in this study to prepare microcapsules with graphene. SMA was similarly selected as an emulsifier. Figure 2 shows the optical morphologies of microcapsules with 2% graphene in shell in different preparation states of the two-step polymerization. The microcapsules forming details can be directly observed through these images. It is well-known that the mean size of microcapsules decreases with the increasing of stirring rate of emulsion in emulsion [3]. To simply this parameter of microcapsules, the stirring rate in this work was pointed as a fixed value of 3000 r·min^−1^. The weight ratio of core/shell was 1/1. In Figure 2a, it can been seen that the core material has been dispersed into droplets and emulsified by hydrolyzed SMA molecules. The mean size of droplets is about 20 μm. Then HMMM prepolymer added in emulsion (Figure 2b). Under a high temperature, the prepolymer has a cross-linking reaction in the first-step polymerization (Figure 2c,d). Core droplets have been encapsulated by HMMM prepolymer molecules in emulsion. After a polymerization process, the graphene and polymer mixture are added in emulsion again. Figure 2e shows the morphologies of the microcapsules with graphene deposition. In the second polymerization, the HMMM/graphene shell have been formed under a high temperature (Figure 2f,g). These microcapsules have regular globe shape with smooth surface without rapture. The dried microcapsules own black color shells. The surface of shells is covered with impurity (Figure 2h). At last, the microcapsules have been washed by alcohol; no adhesion and impurity substance exists on microcapsules (Figure 2i).

**Figure 3:**
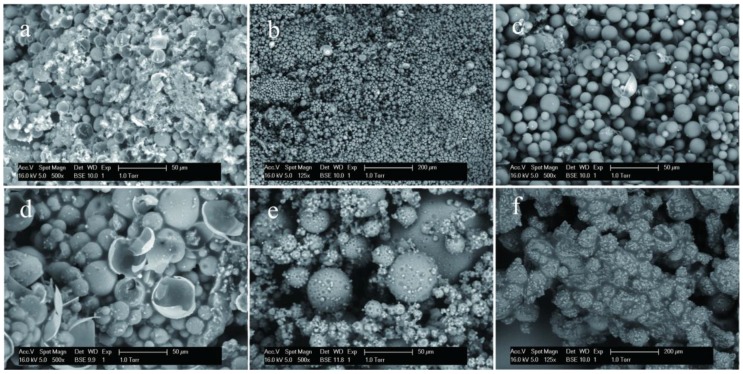
SEM morphologies of self-healing microcapsules with difference contents of graphene in shells, (**a**) 0%; (**b**) 2%; (**c**) 4%; (**d**) 6%; (**e**) 8% and (**f**) 10%.

Figure 3a–f shows the SEM morphologies of microcapsules with 0%, 2%, 4%, 6%, 8% and 10% contents graphene in shell, respectively. In Figure 3a, the morphology of microcapsules without graphene is consistent with the previous results [16,17,43]. Microcapsules have a global shape without rapture. The microcapsules with 2.0–6.0% graphene have a very smooth surface without attachment (Figure 3b,c). The shell thickness and the shell structure can be recognized from the break shells (Figure 3d). On the contrary, the microcapsules with 8.0% and 10.0% graphene have a rough surface and impurity attachment adheres on the shells (Figure 3e,f). In the polymerization process with the same conditions, this phenomenon indicates that not all graphene may be consumed to form shells. The redundant graphene adheres on shells or disperses in emulsion. Based on the SEM morphologies, we can roughly infer that about 6% graphene may be deposits on core droplets to form an organic/inorganic composite structure. At the same time, not all graphene can be absorbed on core droplets because the polymer molecules tangle with graphene during the polymerization. So the unconsumed polymer leads the tangled graphene to be left in emulsion or only adheres incompactly on shells.

In order to measure the shell thickness, microcapsules were mixed in gelatin gel to form composite samples. The dried composites were cut to obtain the cross-section by an ultramicrotome. Table 1 lists the physical structure characters of microcapsules fabricated under emulsion stirring rate of 3000 r·min^−1^. Six types of microcapsules are prepared with different graphene/shell weight percentage of 0%, 2%, 4%, 6%, 8% and 10%, which are noted as MG-0, MG-2, MG-4, MG-6, MG-8 and MG-10, respectively. With the same stirring rate, the microcapsule samples nearly have the same mean size values in a range of 22.4–25.4 μm. Moreover, the microcapsules have the shell thickness value between 2.5 μm and 2.7 μm without a large change. It has reported that the shell thickness is mainly determined by the core/shell weight ratio [38]. To simply the study, all microcapsule samples have a same core/shell ratio of 1/1 in this work. As microcapsule formation in this study is a self-assembly process, the addition of nano-particles may greatly affect the morphology of shells [16]. With the increasing of graphene content, the data indicates that the addition of graphene does not affect the shell thickness. This result is similar to the microcapsules with nano-CaCO_3_/polymer shells [43]. It was found that the nano-CaCO_3_ particles were embedded into polymers to form a stable composite shell structure. Because of the same fabrication mechanism, it may be deduced that graphene has adhered to the core droplets with HMMM to form inorganic/organic composite shells.

### 3.2. Chemical Structure of Microcapsule Shells

**Figure 4:**
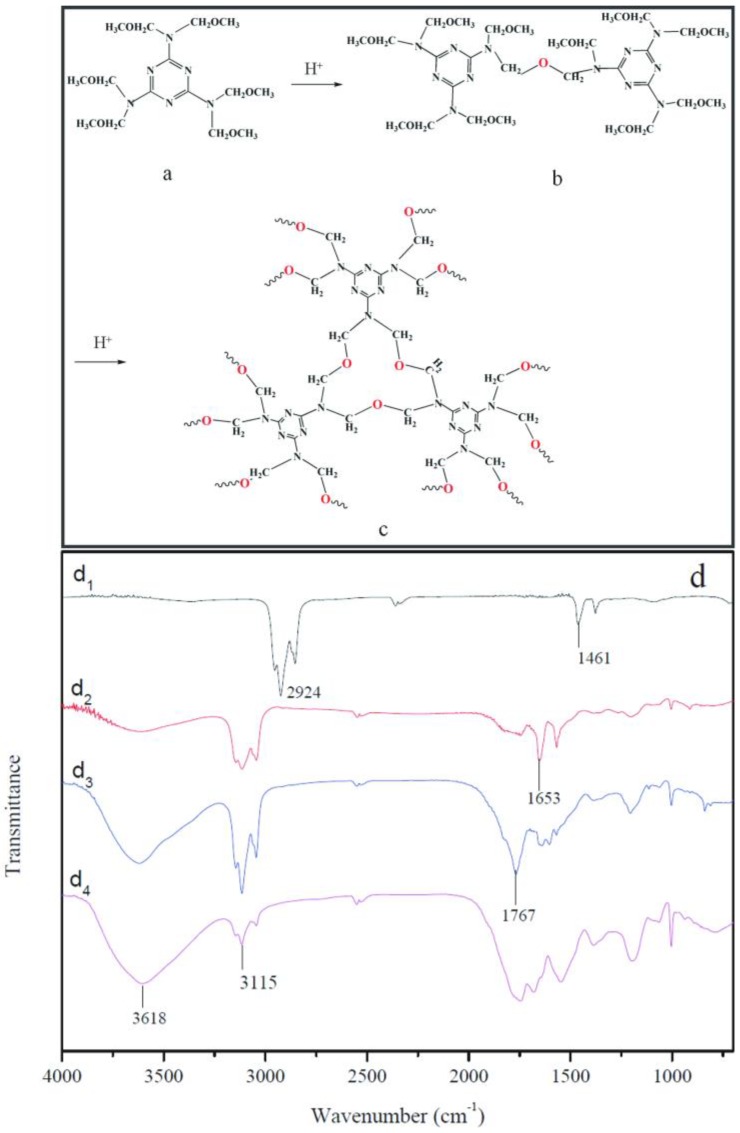
Chemical structure of microcapsule samples, (**a**–**c**) chemical sketch of HMMM polymerization process: (**a**) HMMM prepolymer; (**b**,**c**) the networks of cross-linked HMMM; (**d**) FT-IR spectra of microcapsule samples: (d_1_) rejuvenator, (d_2_) self-healing microcapsules (MG-0) without graphene, (d_3_) self-healing microcapsules (MG-2) with 2% of graphene, and (d_4_) self-healing microcapsules (MG-10) with 10% of graphene.

In this work, HMMM prepolymer has a cross-linked chemical reaction in emulsion state to yield a compactable shell structure. The prepolymer interaction generated oligomer is shown in Figure 4a, b. Under an acid condition, the HMMM prepolymer has a dehydration reaction through methyl oxygen (–OCH_3_) groups. The further cross-linking reactions of the oligomer form the stable shells of microcapsules (Figure 4c). The cross-linked HMMM network provides a protective barrier between the core material and the external environment preventing core material leakage or contamination.

Figure 4d shows the FT-IR spectra (d_1_–d_4_) of rejuvenator, self-healing microcapsules without graphene (MG-0) and self-healing microcapsules with 2% and 10% graphene (MG-2 and MG-10). The spectrum of rejuvenator (d_1_) exhibits the characteristic absorption bands at around 1461cm^−1^ and 2924 cm^−1^ attributed to –CH_3_ asymmetric bending vibration and alkyl −CH_2_ stretching vibration. The spectra of all self-healing microcapsules (d_2_, d_3_ and d_4_) exhibit three absorption bands at around 1653 cm^−1^ (C–C), 3115 cm^−1^ (N–H), and 3618 cm^−1^ (O–H). The characteristic peaks of the rejuvenator no longer exist, which indicates that the rejuvenator has been microencapsulated. The spectra (d_3_) and (d_4_) are microcapsules with different graphene contents in shells. It is found that both samples have O−H bonds, which may belong to the residual water. More graphene in shells does not change the chemical structure of HMMM/graphene composites. In other words, the graphene has a physical bond with cross-linked HMMM networks.

### 3.3. Determination of Graphene Deposition in Shells

**Figure 5:**
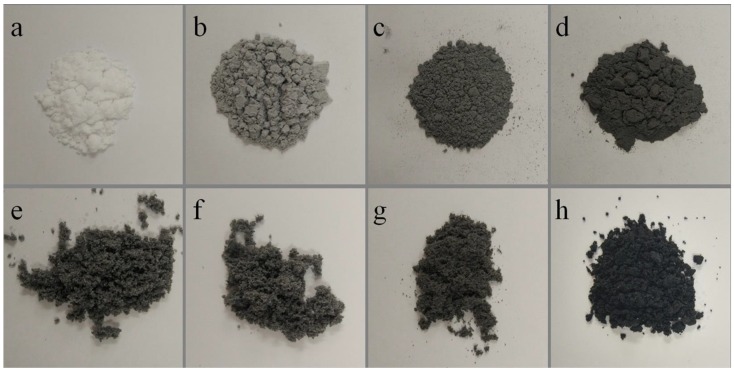
Photographs of self-healing microcapsules without various weight contents graphene in shells, (**a**) 0%; (**b**) 0.5%; (**c**) 1.0%; (**d**) 2.0%; (**e**) 4%; (**f**) 6%; (**g**) 8% and (**h**) 10%.

Figure 5 show the photographs of self-healing microcapsules without various contents graphene of 0%, 0.5%, 1.0%, 2.0%, 4.0%, 6.0%, 8.0% and 10.0%, respectively. In Figure 5a, the color of microcapsules without graphene is white. Graphene has a natural color of black. With the increasing of graphene content in shells, the dark color of microcapsule samples gets deeper and deeper obviously as shown in Figure 5b–h. Through the color changes, it can be deduced that more graphene may have deposited in shells with the increasing of graphene addition. Yet such a deposition or hybridization process has rarely been adopted in emulsion system, little knowledge can be used. Especially, the balance of the electric charge may greatly affected by the graphene additive. It determines the maximum adsorption content of graphene in shells.

**Figure 6:**
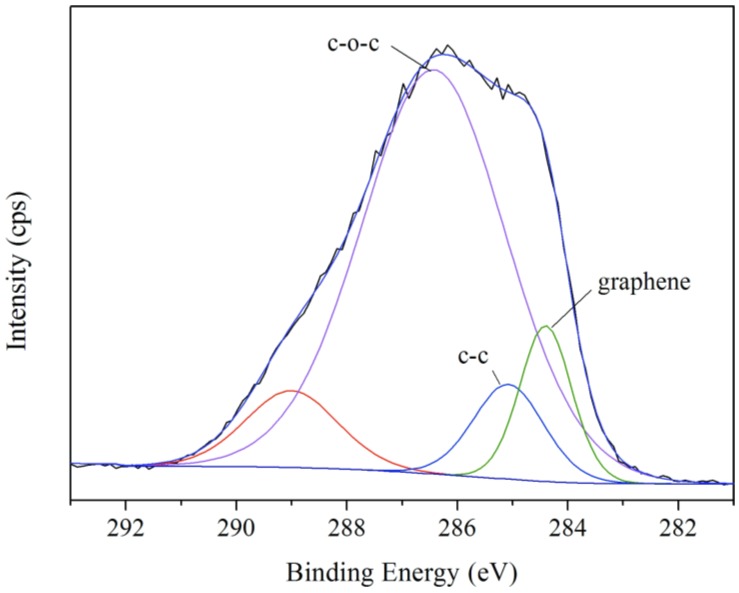
XPS spectra (C1s) of microcapsules with 2% graphene in shells.

XPS can be used to analyze the surface chemistry of a material in its as-received state. XPS is routinely applied to determine the types and the quantity of elements that are present within the top 1–12 nm of the sample surface. Chemical-state analysis is widely used for the element carbon. Chemical-state analysis of the surface of carbon-containing polymers readily reveals the presence or absence of the chemical states of carbon shown in bold, in approximate order of increasing binding energy. For example, the nominal binding energy of the C1s electron is 284.6 eV, subtle but reproducible shifts in the actual binding energy, the so-called chemical shift, provide the chemical state information. In order to further verify the existence of graphene in shells, XPS spectra were analyzed to give more details about the carbon element in microcapsule shells. The ability to produce chemical state information from the topmost few nm of any surface makes XPS a unique and valuable tool for understanding the chemistry of any surface. The local bonding environment of a material is affected by its formal oxidation state, the identity of its nearest-neighbor atom, its bonding hybridization to that nearest-neighbor atom, and in some cases even the bonding hybridization between the atom in question and the next-nearest-neighbor atom. Figure 6 shows the XPS spectra (C1s) of microcapsules with 2% graphene in shells. The curve points out the presence of three functional groups: the nonoxygenated C–C (C–C and C–H, at a binding energy of 285.08 eV), the ether C (C–O, 286.43 eV), the C in the C–N bond (C=N, 289.00 eV). Another C state is found at 284.40 eV, which belongs to the graphene. A similar results has been reported about the binding energy at 284.40 eV assigned to graphene [44]. The XPS results prove that the graphene has deposited on the microcapsules surface. The graphene addition may have an optimal amount to balance both two actions of charge adsorption and chain entanglement. The static electricity balance affected by graphene will be studies in future works.

**Figure 7:**
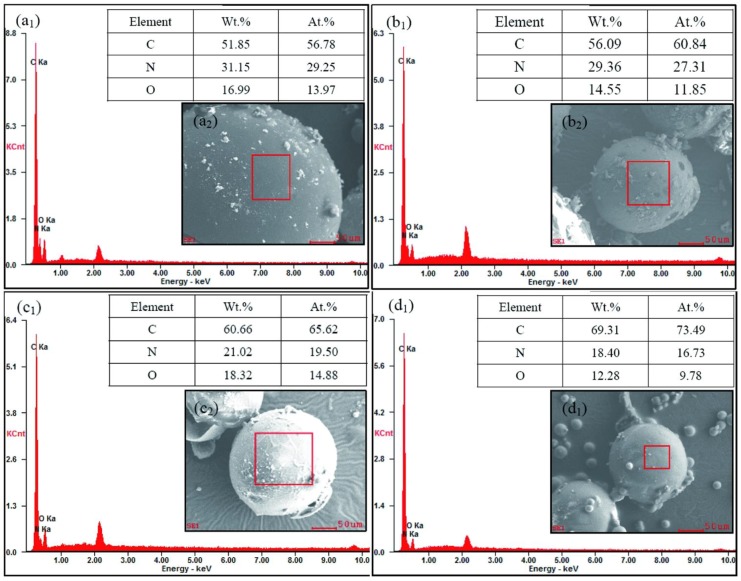
EDS analysis of the self-healing microcapsules without/with graphene, (**a_1_**–**d_1_**) EDS values of MG-2, MG-4, MG-6 and MG-8; (**a_2_**–**d_2_**) SEM morphologies of testing points of MG-2, MG-4, MG-6 and MG-8.

EDS is an analytical technique used for the elemental analysis or chemical characterization of a material. It relies on an interaction of some source of X-ray excitation and a sample. Its characterization capabilities are due in large part to the fundamental principle that each element has a unique atomic structure allowing a unique set of peaks on its electromagnetic emission spectrum. Based on this main principle of spectroscopy, the number and energy of the X-rays emitted from a microcapsule can be measured by an energy-dispersive spectrometer. As the energies of the X-rays are characteristic of the difference in energy between the two shells and of the atomic structure of the emitting element, EDS allows the elemental composition of the microcapsules to be measured. Figure 7a_1_–d_1_ show the EDS results of microcapsule samples (MG-2, MG-4, MG-6 and MG-8) with different graphene in shells. Furthermore, the tables list the weight ratios and atom ratios of elements of C, N and O on the shell surfaces. It has been confirmed that the element measurement points were all on the shells of single microcapsule as the SEM morphologies shown in Figure 7a_2_–d_2_. The presented elements (C, N and O) on the shell were measured. Firstly, it is obvious that the surface carbon contents of the self-healing microcapsules added with graphene are larger values identifying from the C peaks. The characteristic peaks of C indicate the existence of graphene in shells. Secondly, it is found that the weight ratio and atom ratio of N and O both have decreased with the increasing of graphene in shells. For example, the N weight ratios are 31.15%, 29.36%, 21.02% and 18.40% for MG-2, MG-4, MG-6 and MG-8, respectively. In the cross-linking structure of HMMM network, the atom ratio of N/C can be approximated as 1/1 as shown in Figure 4c. Therefore, the atom percentage of C in MG-2, MG-4, MG-6, and MG-8 should be 29.25%, 27.31%, 19.50% and 16.73%, respectively. Then, the atom ratios graphene can be calculated as 27.53%, 33.53%, 46.12% and 56.76% for MG-2, MG-4, MG-6 and MG-8. There is no doubt that graphene can not all deposit in the shells in the reaction. Although this data is not precise, it reflects an increasing trend of graphene contents on shells. This conclusion proves it in another way that the graphene has increased in shells with the increasing of graphene addition during preparation process of microcapsules.

### 3.4. Microstructure of Graphene/Organic Hybrid Shells

**Figure 8:**
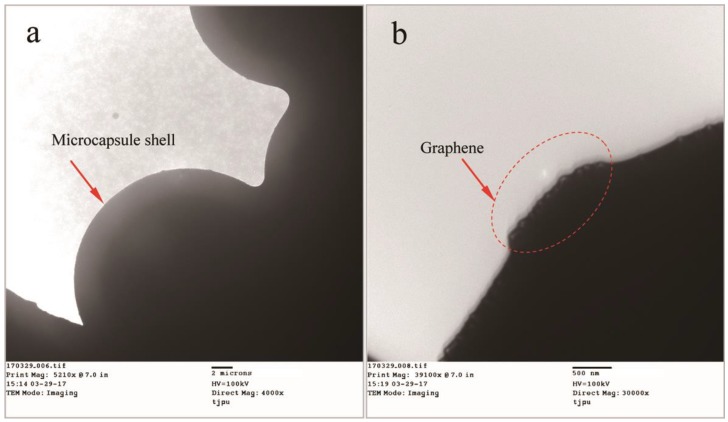
TEM morphologies of microcapsules (MG-2) with graphene/organic structure shells, (**a**) the microcapsules on a copper screen; and (**b**) graphene sheets with a size of 100 nm extended out of a microcapsule shell surface.

Besides the determination of graphene deposition in shells, the microstructure of graphene/organic hybrid shells needs to be investigated. The knowledge is helpful to understand the relationship between the graphene states in shells and the physical properties of shells. For example, the thermal properties and mechanical properties of microcapsules may be greatly influenced by the graphene/organic hybrid structure shells. Figure 8 shows the TEM morphologies of microcapsules with graphene/organic hybrid structure shells. In Figure 8a, microcapsules can be clearly discriminated on a copper screen through their global shape. It can be seen from an enlarged TEM morphology as shown in Figure 8b that some particles adhere on the shell surface as the arrows point. Before the TEM tests, all the microcapsules had been washed to remove the impurity substance and unconsumed materials including the polymer and graphene. Therefore, the particles on the shell can only be attributed to the existence of graphene. The arrows point a sheet structure with a size of 100 nm extending out of a microcapsule shell surface. This shape and size of the sheet is consistent to the monolayer structure of graphene.

**Figure 9:**
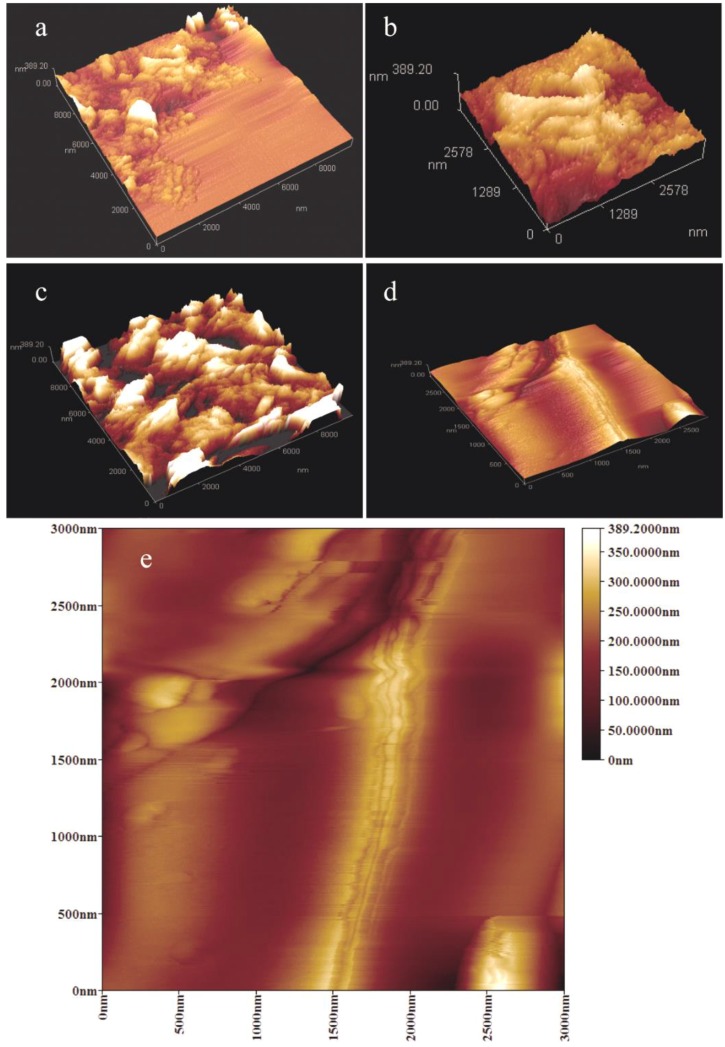
AFM morphologies of single microcapsule surface with/without graphene/organic hybrid structure shells, (**a**) a microcapsule (MG-0) surface without graphene; (**b**–**d**) microcapsule samples of MG-2, MG-4 and MG-6; and (**e**) an enlarged AFM morphology of MG-6.

Figure 9 shows the AFM morphologies of single microcapsule surface with/without graphene. In Figure 9a, the shell without graphene has a relative rough surface. Comparatively, graphene can be seen on the shell surfaces as shown in Figure 9b–d, which lead the surfaces to a smooth surface structure. This result is agreement with the SEM analysis. Moreover, the lamellar structure of graphene can be recognized. All the lamellar structure has a size less than 200 nm, which coincides with the size of graphene. At the same time, it can be found that with the increasing of graphene addition (MG-2, MG-4 and MG-6), more graphene has been appeared in shells. This conclusion is consistent with previous results. It must be noted that some graphene layers may pile together because of the electrostatic attraction. Despite using ultrasonic dispersion technique in this work, the graphene is hard to be dispersed as an absolute uniform state in emulsion system. This problem also occurs in other polymer systems with nano-particles additive [25]. More details of graphene states in shell can be obtained from an enlarged AFM morphology of MG-6. The graphene sheet structure can be observed clearly as the arrows pointing. The sheets disperse in cross-linked HMMM forming an inorganic/organic composite.

### 3.5. Thermal Stability of Microcapsules

**Figure 10:**
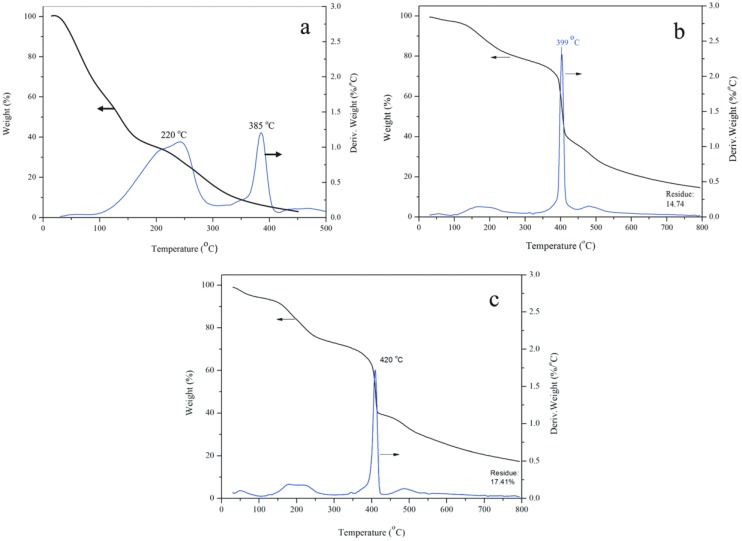
TG/DTG curves of the self-healing microcapsules without graphene, (**a**) MG-0; (**b**) MG-2; and (**c**) MG-6.

Figure 10 shows the TG/DTG (derivative thermogravimetric analysis) curves of the self-healing microcapsule samples of MG-0, MG-2 and MG-6. Firstly, it can be seen from Figure 10a, the self-healing microcapsules without graphene starts to lose weight at about 120 °C and completes at about 400 °C. The weight losing processes of microcapsules contains two steps as shown in its DTG curve. In the first step, the weight of microcapsules decreases sharply from 132 °C, which is due to the fact that residual micro-molecular organics in microcapsules is lost through drying. At the same time, the microcapsules have broken and released the oily small molecules of rejuvenator with the increasing of temperature. Then the rejuvenator has decomposed rapidly. The second step happens in a temperature range between 393 °C and 473 °C, which is caused by the decomposition of microcapsules shells.

Comparing to MG-0, the beginning weight loss rate of MG-2 has obvious decreased as shown in Figure 10b, which implies that its thermal stability has been improved. Before the temperature of 385 °C, the weight loss curve has a small slope. It means that the shells still keep a compactable structure without rapture. The weight loss is about 15% before 200 °C can be attributed to the residual water of small molecules in shells. The DTG curve has a peak at 399 °C, which can be considered as the moment that the most of microcapsules have broken and released the rejuvenator. Under such a high temperature, the rejuvenator molecules have decomposed rapidly. The reason for the above phenomena can be attributed to two facts. One is that the graphene has a good heat transfer performance and makes the heat distribution in the microcapsules more uniform, thereby improving the thermal stability of microcapsules. The loss weight curve is nearly horizontal at about 800 °C, which points that all organic materials have been decomposed. Secondly, the graphene/HMMM composites structure can enhance the thermal stability of microcapsules. Similar result has already been reported in previous works about other microcapsules with nano-particles/polymer shells [23,27]. Figure 10c shows the TG/DTG curve of the self-healing microcapsule sample of MG-6. Its TGA curve has a similar shape with MG-2. But its DTG curve has a peak at 420 °C, which is higher than the 399 °C peak temperature of MG-2. This phenomenon means that most of microcapsules have broken and released the rejuvenator at an even higher temperature. More graphene can enhanced the thermal stability of microcapsules. The MG-6 sample has a residual testing weight about 17.41% at 800 °C. The final residual weight belongs to the total weight of organic carbonization and graphene. In the TG/DTG tests, the MG-2 and MG-6 have an equal weigh. Therefore, the increasing of residual weight is attributed to the increasing of graphene in samples.

### 3.6. Thermal Conductivity Analysis

**Figure 11:**
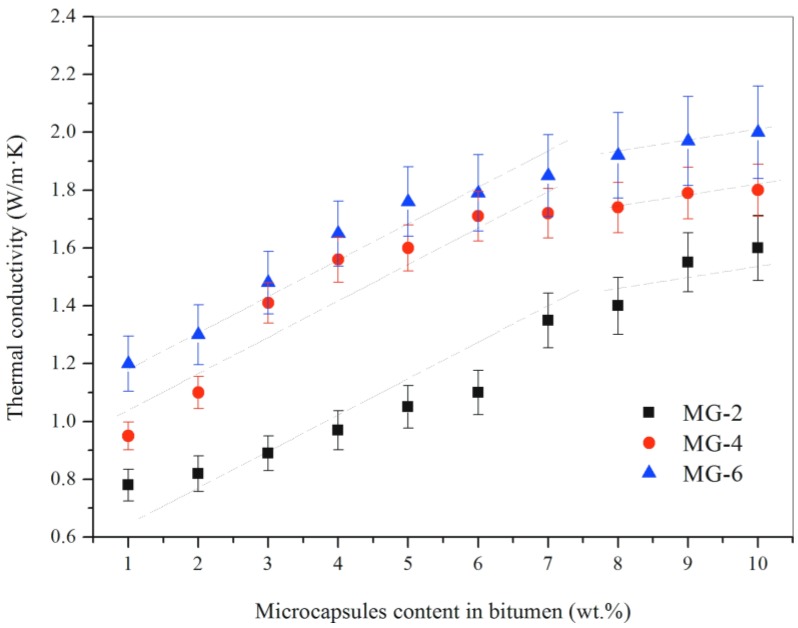
Thermal conductivity of bitumen samples with various weight contents (1–10%) of graphene microcapsules (MG-2, MG-4 and MG-6).

Thermal conductivity is an important parameter reflecting the thermal transmission efficiency of materials. A higher thermal conductivity value means that a material has a relative faster heat absorbing or releasing capability during temperature changes. Figure 11 shows the thermal conductivity of bitumen samples with various contents of microcapsules (1–10%). There types of microcapsule samples were used in this study (MG-2, MG-4 and MG-6). For the bitumen sample with the same microcapsules, its thermal conductivity increases with the increasing of microcapsules content in bitumen. For example, the thermal conductivity of bitumen sample with MG-2 changes from 0.81 to 1.57 W/(m·K) when the microcapsules content enlarges from 1% to 10%. In another hand, the bitumen samples with the same content microcapsules do not have the same thermal conductivity values. The conductivity values of bitumen samples have increased with the increasing of graphene contents in shells. For example, the bitumen samples with 1% microcapsules (MG-2, MG-4 and MG-6) have the thermal conductivity values of 0.81, 0.96 and 1.25 W/(m·K).

In Figure 11, the thermal conductivity values of the bitumen sample with the same microcapsule have a sharply liner increase with the increasing of microcapsule contents from 1% to 7%. The thermal conductivity of MG-2/bitumen has increased approximately 160%. However, its thermal conductivity increases gently to about 1.57 W/(m·K) with the microcapsules contents changing to 10%. Similarly, the thermal conductivity of MG-4/bitumen or MG-6/bitumen has a level liner trend when the microcapsule content changes from 7% to 10%. All the above evidence indicate that a little amount of graphene can improve the thermal conductivity of bitumen significantly. It can be attributed to the excellent thermal conductivity of graphene microcapsule shells. It can also be found that the thermal conductivity does not increase greatly, even though more graphene has been added. In other words, the graphene in shells, unlike pure graphene, has a limitation to increase the thermal conductivity of bitumen. As it is well-known that a polymer is a poor heat conductor, the inorganic additive can significantly enhance its thermal conductivity. It can be imagined that the graphene overlaps will be the perfect microstructure creating heat transition bridges, which has the lowest thermal transition barrier. When the bridges have been formed, more microcapsules addition can not increase the thermal conductivity effectively. Future works will be carried out to optimize the microstructure of graphene/organic hybrid shells to understand more details of heat conductivity.

### 3.7. Electric Conductivity Analysis

Electrical resistivity is an intrinsic property that quantifies how strongly a given material opposes the flow of electric current. A high electrical resistivity value means a material that not allows the flow of electric current. Most of polymers have a high electrical resistivity, which are considered as insulators. Bituminous materials also are insulators without conductivity. It is crucial in this study that bitumen samples have certain conductivity, and they can convert electric energy into heat energy. A literature review shows that the conductivity of organic materials can be enhanced by the addition of graphene with several orders of magnitude [45,46]. For this purpose, several tests had been carried out to evaluate the effects of graphene addition on the electric conductivity of bituminous materials.

**Figure 12:**
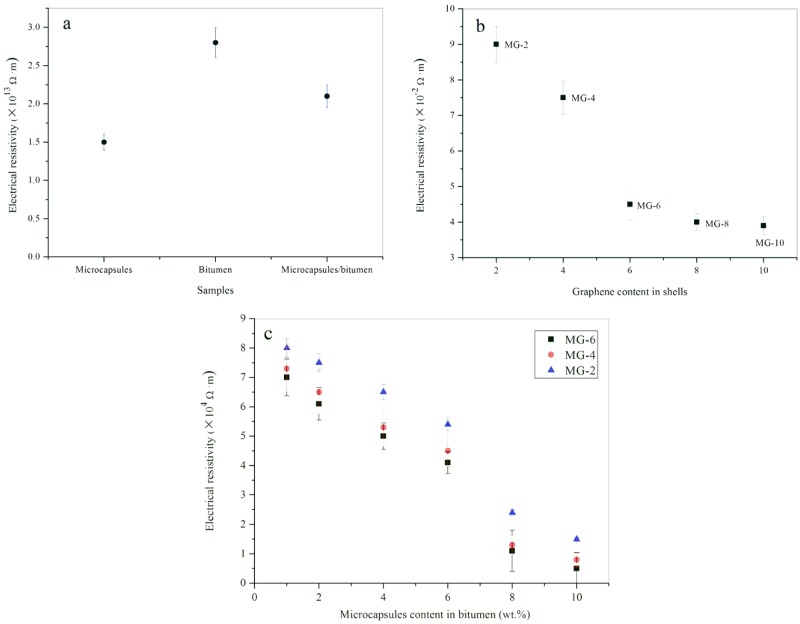
Electrical resistivity of microcapsules and bitumen samples, (**a**) MG-0, pure bitumen, and bitumen with 10 wt % MG-0; (**b**) microcapsule powder samples of MG-2, MG-4, Mg-6, MG-8 and MG-10; and (**c**) bitumen/microcapsule composite samples, MG-2, MG-4 and MG-6 in bitumen with weight content of 1%, 2%, 4%, 6%, 8% and 10%.

Figure 12a shows the three electrical resistivity values of MG-0, pure bitumen, and bitumen with 10 wt % MG-0. Pure bitumen has a high electrical resistivity of 2.8 × 10^13^ Ω·m. Even mixing with microcapsules (1.51 × 10^13^ Ω·m), the bitumen/microcapsule composite sample still has an electrical resistivity value of 1.25 × 10^13^ Ω·m. As its components are polymeric materials, it still can be considered as a insulator without conductivity. Figure 12b shows the electrical resistivity values microcapsule powder samples of MG-2, MG-4, Mg-6, MG-8 and MG-10. MG-2 has the maximum value of 9.0 × 10^−2^ Ω∙m and MG-10 has the minimum value of 3.8 × 10^−2^ Ω·m. With the increasing of the weight content of graphene in shells, the microcapsule samples has a sharp decrease in electrical resistivity. Comparing to pure microcapsules without graphene, the microcapsules with graphene has reduced the electrical resistivity of about fifteen orders of magnitude. Pure graphene has a electrical resistivity value about 9.0 × 10^−8^ Ω·m [19]. As a powder material, the microcapsule particles contacts with each other, this also allows the graphene in adjacent microcapsules to contact with each other. Therefore, a conductive pathway has formed. Because of the influence of HMMM, the graphene in shells cannot have the conductivity of pure graphene.

Figure 12c shows the electrical resistivity values of microcapsule/bitumen composite samples with microcapsule weight contents of 1%, 2%, 4%, 6%, 8% and 10%. Three types of microcapsule (MG-2, MG-4 and MG-6) were selected for this investigation. For the MG-2/bitumen samples, their electrical resistivity values have a linear decrease with the increasing of microcapsule contents in bitumen from 1% to 6%. More microcapsules addition can decrease the electrical resistivity values of bitumen samples. However, the rate of decrease has shrunk by comparing the slopes of the fitted curves. Bitumen samples mixing with MG-2 and MG-4 both have the same trend. These phenomena may imply that the conductive path needs to be set up with enough microcapsules. After the forming of graphene bridges, the electrical resistivity will not allowed to be decreased sharply because of the insulated nature of bituminous materials.

## 4. Conclusions

An electrothermal self-healing method had been reported in this study by using microcapsules containing rejuvenator with graphene/organic hybrid structure shells. These microcapsules had been fabricated by a two-step self-assembly polymerization process. With the help of emulsified SMA molecules, prepolymer HMMM and graphene deposited on oily rejuvenator droplets forming compact shells. Electric charges were the driving force of the deposition. These microcapsules combined the advantages and avoiding the disadvantages of the heat induction and rejuvenator self-healing for asphalt material. The microcapsules had electric conductivity capability because of the advent of graphene. Experimental tests were carried out to characterize the physicochemical properties of microcapsule samples and microcapsules/bitumen composite samples. The following conclusions can be drawn:

(1) Morphology observations showed that the microcapsules with graphene had a global shape with a compact shell structure. The microcapsules with 2.0–6.0% graphene had a very smooth surface without attachment. The polymer molecules tangled with graphene during the polymerization, so the unconsumed polymer made the tangled graphene to be left in emulsion or only adhere incompactly on shells.
(2) Chemical structure analysis confirmed that the HMMM prepolymer had a cross-linked chemical reaction in emulsion state to yield a compactable shell structure. Graphene did not change the chemical structure of HMMM/graphene composites, it only had a physical bond with cross-linked HMMM networks.
(3) The results proved that the graphene had deposited on the microcapsules surface. The graphene addition had an optimal amount to balance both two actions of charge adsorption and chain entanglement. It was found that the microcapsules had an increasing trend of graphene contents on shells with the increasing of graphene addition during preparation process of microcapsules.
(4) The graphene/HMMM composites structure enhanced the thermal stability of microcapsules. Moreover, the graphene had a good heat transfer performance and made the heat distribution in the microcapsules more uniform, thereby improving the thermal stability of microcapsules.
(5) The thermal conductivity of microcapsules sharply increased to about 8.0 W/m^2^·K with only 2.0 wt % graphene in shells. At the same time, electrical resistivity of microcapsules/bitumen samples had a decreased trend with more graphene in bitumen.

## Figures and Tables

**Table 1:** Characters of microcapsules with various graphene contents in shells.

Microcapsules Sample	Core/Shell Weight Ratio	Graphene /Shell (wt %)	Emulsion Rate (r·min^−1^)	Mean Size (μm)	Shell Thickness (μm)
MG-0	1/1	0	3000	22.4	2.5
MG-2	1/1	2	3000	22.5	2.4
MG-4	1/1	4	3000	22.9	2.5
MG-6	1/1	6	3000	23.4	2.6
MG-8	1/1	8	3000	24.3	2.6
